# Differential effects of vitamin D2 and D3 supplements on 25-hydroxyvitamin D level are dose, sex, and time dependent: a randomized controlled trial

**DOI:** 10.1186/s12902-017-0163-9

**Published:** 2017-02-24

**Authors:** Muhammad M. Hammami, Ahmed Yusuf

**Affiliations:** 1 Clinical Studies and Empirical Ethics Department, King Faisal Specialist Hospital and Research Center, P O Box # 3354 (MBC 03), Riyadh, 11211 Saudi Arabia; 2 Alfaisal University College of Medicine, Riyadh, Saudi Arabia

**Keywords:** Cholecalciferol supplement, Ergocalciferol supplement, Sex effect, Dose effect, D2 level, D3 level, 25-hydroxyvitamin D2 level, 25-hydroxyvitamin D3 level, Creatinine production, BMI

## Abstract

**Background:**

Vitamin D (D) supplements are indispensable for its world-wide deficiency. Controversy continues on ergocalciferol (D2) and cholecalciferol (D3) relative potency as well as on dosing-schedule and sex role in raising 25-hydroxy D (25(OH)D) level, the best indicator of D status.

**Methods:**

We randomized 279 adults to daily D2, D3, D2/D3, or placebo; 2-weekly D2 or D3; or 4-weekly D2 or D3 (250,000 IU over/140 days). Randomization sequence, stratified by body-mass-index (BMI) and sex, was concealed from study coordinators and participants who were then blinded to capsules’ content. D2, D3, 25(OH)D2, and 25(OH)D3 Serum levels were determined blindly on days 0,1,2,3,4,7,14, and 2-weekly thereafter by high performance liquid chromatography assay. The results of 269 participants were available for analysis. Primary endpoint was area-under-the-curve (AUC) of 25(OH)D (25(OH)D2 + 25(OH)D3) adjusted for sex, BMI, and baseline 25(OH)D level.

**Results:**

Mean(SD) age was 33.0(8.5) year, 41% were males, and 85% completed follow-up. Baseline 25(OH)D level was 39.8(11.9) and increased by 3.3(11.6) and 28.6(16.3) nmol/L, in the placebo and active-treatment groups, respectively. AUC from day 0 to 140 (AUC_140_) of 25(OH)D was 40% (D3 daily) to 55% (D3 2-weekly) higher with active-treatment than placebo (*p* < 0.001). 25(OH)D2 AUC_140_ was higher in daily than 2-weekly (17%, *p* = 0.006) and 4-weekly (20%, *p* = 0.001) D2-treated groups. 25(OH)D3 AUC_140_ was lower in daily than 2-weekly (11%, *p* = 0.002) and 4-weekly D3-treated groups (10%, *p* = 0.008). In D2-treated groups, there was 16.4 nmol/L decrease in 25(OH)D3 level that correlated (*p* < 0.001) with 25(OH)D2 level increase (*r* = 0.48) and baseline 25(OH)D level (*r* = 0.58), in one participant with measurable baseline 25(OH)D2 level, D3 caused a similar decrease in 25(OH)D2 level, while in the D2/D3-treated group, 25(OH)D3 level didn’t increase. Incremental AUC from day 0 to 7 (AUC_7_) of D3 and 25(OH)D3 in D3-treated groups were 118–243% higher and 31–39% lower, respectively, than incremental AUC_7_ of D2 and 25(OH)D2 in D2-treated groups. Incremental AUC_7_ of D3 and 25(OH)D3 in D3-treated groups and D2 and 25(OH)D2 in D2-treated groups were higher in females than males (55, 13, 64, and 28%, respectively). Baseline 25(OH)D level predicted response to D2 and D3 (*p* < 0.001), whereas, BMI was significant predictor only for early response to D2.

**Conclusions:**

Effects of D2 and D3 supplements on 25 (OH)D level may be dosing-schedule and sex-dependent. D2-associated reduction in 25(OH)D3 level may be related to total 25(OH)D level rather than being D2-specific. D2 may be 25-hydroxylated faster than D3.

**Trial registration:**

ClinicalTrial.gov identifier: NCT01170494 (registered July 25, 2010).

## Background

The increasingly recognized effects of vitamin D (D) on skeletal and extra-skeletal tissues [1, 2] together with worldwide D deficiency have amplified attention to D supplementation. Nevertheless, the relative potency of ergocalciferol (D2) vs cholcalciferol (D3), and the preferable dosing strategy continue to be controversial [3].

The same unit is used for both D2 and D3, suggesting biological equivalence in terms of raising serum total 25-hydroxy D (25(OH)D) level, [4] the best current biomarker of D status. However, published studies have yielded mixed results [4–13]. This may be due in part to the facts that supplement-induced increase in 25(OH)D level may be related to baseline level, [9, 11, 13–16] body mass index (BMI), [12–14, 16–21] sex, [13, 22, 23] dosing strategy, [24] meal content, [25] and duration of follow up, which have not been systematically controlled for. A meta-analysis found that D3 is significantly more potent than D2 as bolus dosing but not as daily dosing [24]. Further, the dose–response curve may be curvilinear rather than linear [13, 20, 21, 26, 27].

D2 and D3 differ in their side chain structure, conversion to 25(OH)D by hepatic 25-hydroxylase, affinity (and affinity of their metabolites) to circulating D binding protein, inactivation by 24-hydroxylation, and plasma half-life [24, 28]. However, in respect to binding to D receptor, the active dihydroxyvitamin D forms, 1,25(OH)_2_D2 and 1,25(OH)_2_D3, appear to be comparable [1]. Further, 25(OH)D2 and 25(OH)D3 appear to be equally recognized by the kidney 1-alpha hydroxylase [29–31].

Equivalent oral doses of D given daily compared to less frequently may result in differing increments in 25(OH)D [32] and D [2] levels. Circulating D has better general cellular accessibility than 25(OH)D due to its lower affinity to circulating D binding protein and may play an important physiological role as a substrate for many tissues [2].

It has been noticed that D2 supplementation is associated with reduction in 25(OH)D3 [5, 8, 33–35] and 1,25(OH)_2_D3 [36] levels. The underlying mechanism has not been elucidated; it is possible that the reduction is not specific to D2 supplementation and merely reflects a response to increasing 25(OH)D levels.

The primary aim of this study was to systematically evaluate the relative efficacy of various dosing strategies of D2 and D3 oral supplements in raising 25(OH)D levels.

## Methods

### Design

The study was randomized, placebo-controlled, partially blinded trial to compare the effect of seven D oral regimens on 25(OH)D level. Participants were randomly allocated to daily D2, D3, combination of D2 and D3, or placebo; 2-weekly D2 or D3; or 4-weekly D2 or D3. Total D dose in the active treatment groups was 250,000 IU over 140 days.

### Participants

Volunteers were recruited via advertisement throughout the King Faisal Specialist Hospital and Research Center (KFSH&RC) and other medical centers in the City of Riyadh, Saudi Arabia. We enrolled healthy non-pregnant adults (age 18–60 years) living in Riyadh area who don’t consume more than one serving of milk daily, don’t take vitamin supplements, habitually have less than 10 h of sun exposure weekly, don’t suffer from granulomatous, liver, or kidney diseases, don’t take anticonvulsants, barbiturates, or steroids, and have 25(OH)D level between 20 and 50 nmol/L. Potential participants were screened by obtaining medical history and the following tests: complete blood count, serum creatinine, calcium, phosphorous, albumin, bilirubin, and alanine aminotransferase, and spot urine calcium, phosphate, and creatinine. The study was conducted at KFSH&RC from February 2013 through April 2016 after obtaining approval of the KFSH&RC Research Ethics Committee. All participants gave written informed consent and were compensated based on the Wage-Payment model [37] in a prorated manner.

### Procedures and interventions

Ergocalciferol and cholecalciferol crystals (40,000,000 IU/g) were purchased from AGD Nutrition, LLC (Lewisville, TX, USA) and manufactured together with a matching placebo by Jamjoom Pharma (Jeddah, Saudi Arabia) into green soft gelatin capsules containing 2000, 25,000, or 50,000 IU of either D2 or D3 or a combination of 1000 IU D2 and 1000 IU D3. The content of the capsules was confirmed by in-house laboratory analysis (Jamjoom Pharma, accuracy 98–102%, coefficient of variation (CV) <2%) at manufacturing date and yearly thereafter. On average, the recovered content ranged from 89.33% (D3 2000 IU) to 91.91% (D2 25,000 IU) of the label claim and showed no trend of decrease over study period.

Daily doses (D2 2000 IU, D3 2000 IU, combined D2 1000 IU and D3 1000 IU, or placebo) on days 0, 1, 2, 3, 4, 7, and 14 and 2-weekly thereafter and all of the 2-weekly (D2 25,000 IU or D3 25,000 IU) and 4-weekly (D2 50,000 IU or D3 50,000 IU) doses were administered by study coordinators in the research clinic after blood samples were obtained and a standardized meal was given. The rest of the daily doses were dispensed to participants on a 2-weekly basis to self-administer with the first meal of the day; compliance was emphasized and checked by capsule counting at each research clinic visit. Participants on daily doses were asked to skip the dose on three Saturdays every 4 weeks so that their 4-weekly dose totals 50,000 IU. All participants were asked to report any more than habitual sun exposure or new medication/supplement. All of the seven active-treatment groups were given the same total D dose (250,000 IU over 140 days) and followed up for 140 days.

D2, D3, 25(OH)D2, and 25(OH)D3 serum levels were simultaneously and blindly measured by a locally validated reversed-phase high performance liquid chromatography assay (HPLC) [38] on days 0, 1, 2, 3, 4, 7, and 14, and 2-weekly thereafter. The intra-assay and inter-assay CVs were, respectively, 4.9 and 7.5% for D2, 4.8 and 6.0% for D3, 5.0 and 6.7% for 25(OH)D2, and 6.3 and 6.9% for 25(OH)D3. Limits of detection and quantification were, respectively, 7.5 and 12.5 nmol/L for D2 and D3 and 5 and 12.5 nmol/L for 25(OH)D2 and 25(OH)D3 [38]. Serum calcium and phosphorous and spot urine calcium, phosphate, and creatinine levels were determined by the clinical laboratory at KFSH&RC on days 0 and 140.

### Randomization and blinding

Blocked (block size = 8) randomization sequences, stratified by body mass index (BMI ≤ 30, ≥30 kg/m^2^) and sex were generated (by MMH) using an online program [39]. Assignment was concealed from potential participants and recruiting coordinators. Participants and study coordinators continued to be blinded to the content of individual assignments (D2, D3, D2 and D3, or placebo for daily doses; D2 or D3 for 2-weekly and 4-weekly doses).

### Outcome measures and analysis

The primary outcome measure was the area-under-the-curve from day 0 to 140 (AUC_140_) of 25(OH)D. Predetermined secondary outcome measures were AUC_140_ of 25(OH)D2, 25(OH)D3, D2, and D3 as well as incidence of hypercalcemia and hypercalciuria. AUC from day 0 to 7, day 0 to 14 and day 0 to 28 (AUC_7_, AUC_14_, and AUC_28_) were also calculated. AUCs were analyzed using analysis of covariance (ANCOVA). The model included treatment group, BMI, sex, and the corresponding day zero level. One way ANOVA was used to compare baseline continuous variables among groups and *t* test was used to compare day zero and day 140 continuous variables as well as estimated effect sizes. Analyses were performed (by MMH) with IBM SPSS Statistics version 21 software. Two-tailed *p*-values and 95% confidence intervals (CI) are reported.

## Results

**Fig. 1:**
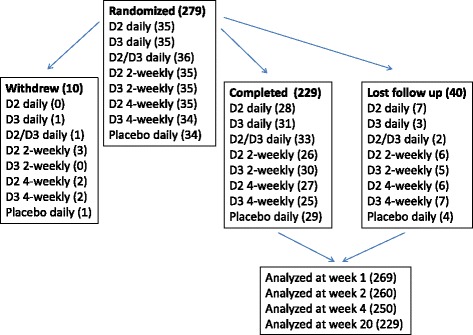
Participants flow chart. “Withdrew” indicates participants who did not complete the first week of the study and who were excluded from analysis

Two hundred seventy nine participants were randomized to 8 groups (D2 daily, D3 daily, combined D2/D3 daily, placebo daily, D2 2-weekly, D3 2-weekly, D2 4-weekly, or D3 4-weekly). Ten participants withdrew within the first week of the study and were not included in the analysis. Two hundred twenty nine participants completed the study and 40 (15%) lost to follow up, including one who became pregnant (Fig. 1).

Table 1 summarizes the main characteristics per treatment group of the 229 participants who completed the study and of the entire cohort of 269 participants. There were no statistically significant differences among the eight groups in the listed characteristics (*p* = 0.32–0.90 for the 229 participants, *p* = 0.41–0.90 for the entire cohort). The 269 participants had a mean (SD) age of 33.0 (8.5) years and baseline 25(OH)D level of 39.8 (11.9) nmol/L, and 41% were males. All were of Middle Eastern or East Asian ethnicity.

**Table 1:** Baseline characteristics of study participants

	D2 daily	D3 daily	D2/D3 daily	D2 2-weekly	D3 2-weekly	D2 4-weekly	D3 4-weekly	Placebo daily
Number	28	31	33	26	30	27	25	29
	35	34	35	32	35	33	32	33
Age, year	34.4 (10.2)	34.5 (9.7)	33.1 (6.8)	31.5 (6.1)	33.5 (10.5)	32.3 (7.2)	32.6 (8.5)	32.8 (7.3)
	34.7 (9.4)	33.7 (9.7)	32.8 (7.1)	31.5 (7.8)	33.4 (10.5)	33.5 (8.0)	31.4 (8.1)	32.4 (7.3)
Male, number (%)	13 (46)	13 (42)	15 (45)	12 (46)	13 (43)	11 (41)	10 (40)	13 (45)
	14 (40)	14 (41)	14 (40)	14 (44)	14 (40)	13 (39)	14 (44)	13 (39)
BMI, kg/m^2^	24.6 (2.9)	26.1 (5.1)	26.2 (3.8)	24.1 (4.0)	24.8 (5.1)	25.3 (5.2)	25.6 (3.4)	23.9 (4.3)
	25.3 (3.3)	25.9 (4.9)	26.0 (3.6)	24.3 (3.9)	24.8 (4.7)	25.4 (4.8)	25.9 (3.7)	24.1 (4.4)
Sun exposure, hour/week	2.0 (2.2)	2.2 (2.1)	2.3 (2.3)	2.3 (2.7)	3.0 (2.9)	2.5 (2.5)	3.2 (3.1)	2.3 (2.3)
	2.3 (2.6)	2.2 (2.0)	2.3 (2.3)	2.7 (3.0)	2.8 (2.7)	2.4 (2.5)	3.1 (3.0)	2.2 (2.2)
Serum 25(OH)D, nmol/L	39.5 (12.2)	41.3 (10.7)	40.7 (14.5)	38.2 (10.5)	39.5 (12.5)	40.9 (12.0)	40.9 (12.2)	42.9 (10.2)
	38.8 (12.1)	40.9 (10.3)	40.8 (14.5)	37.6 (10.5)	39.4 (13.0)	39.5 (12.7)	39.4 (12.3)	41.8 (10.3)
Serum 25(OH)D3, nmol/L	39.1 (12.1)	41.3 (10.7)	38.0 (12.1)	38.2 (10.5)	38.0 (11.5)	40.4 (12.2)	40.9 (12.2)	42.9(10.2)
	37.2 (12.3)	40.3 (10.9)	37.8 (12.3)	37.6 (10.5)	38.2 (12.2)	38.7 (12.8)	39.4 (12.3)	41.8 (10.3)
Urinary calcium/creatinine, mol/mol	0.30 (0.19)	0.39 (0.21)	0.32 (0.22)	0.33 (0.19)	0.37 (0.26)	0.42 (0.24)	0.32 (0.20)	0.40 (0.24)
	0.34 (0.23)	0.39 (0.20)	0.29 (0.17)	0.32 (0.18)	0.37 (0.26)	0.38 (0.24)	0.31 (0.20)	0.38 (0.23)
Urinary phosphate, creatinine, mol/mol	1.69 (0.80)	1.83 (1.02)	1.57 (0.91)	1.70 (0.67)	1.75 (0.76)	2.08 (1.74)	1.47 (0.78)	1.79 (1.06)
	1.66 (0.83)	1.79 (1.01)	1.53 (0.78)	1.62 (0.66)	1.81 (0.74)	2.00 (1.59)	1.60 (1.18)	1.68 (1.04)

Data are means (SD), unless indicated otherwise. The first raw of each entry describes the 229 participants who completed the study; the second raw describes the entire cohort of 269 participants. BMI, body mass index. 25(OH)D, total 25-hydroxyvitamin D. 25(OH)D3, 25-hydroxyvitamin D3

Incompliance with self-administered daily doses as determined by capsule counting was 1.6% for the D2, 1.3% for the D3, 1.0% for the combined D2/D3, and 1.3% for the placebo group (9,8,7, and 6 participants missed a total of 56, 52, 40, and 46 doses, respectively). None of the participants reported more than habitual sun exposure or taking supplements containing vitamin D during enrollment. At day 140, there was no incidence of hypercalcemia and mean (SD) changes in urinary calcium/creatinine ratio and phosphate/creatinine ratio in the seven active-treatment groups were 0.013 (0.259) and −0.183 (1.164) mol/mol, respectively (*p* = 0.48 and *p* = 0.03, respectively). In the placebo group, they were −0.061 (0.270) and −0.236 (1.035) mol/mol, respectively. These changes were the results of significant increase in urinary calcium and creatinine levels with a mean (95% confidence interval, CI) of 0.90 mmol/L (CI, 0.34 to 1.47, *p* = 0.002) and 1.75 mmol/L (CI, 0.56 to 2.94, *p* = 0.004), respectively, and insignificant increase in urinary phosphate of 0.37 mmol/L (−2.11 to 2.85, *p* = 0.77) in the 7 active treatment groups. There were no significant changes in the corresponding parameters in the placebo group (*p* = 0.23 to 0.95). No adverse events were reported.

### Differential effects of vitamin D regimens on 25(OH)D level

**Fig. 2:**
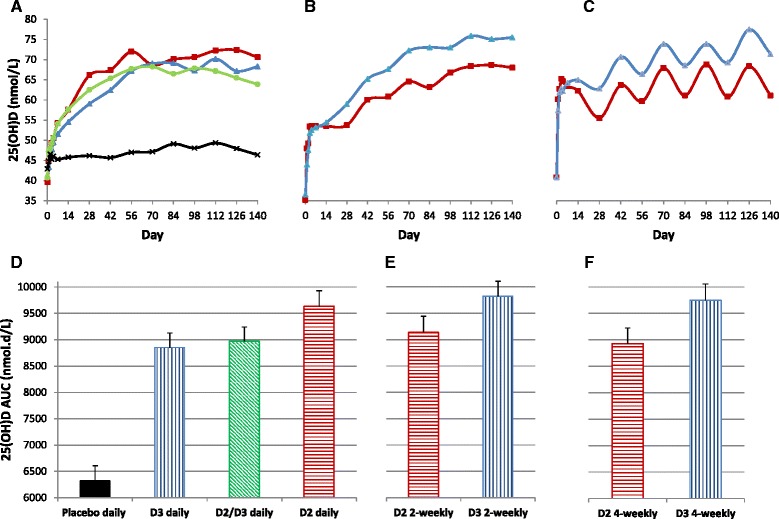
**a**, **b**, and **c**, data represent mean total 25-hydroxyvitamin D (25(OH)D) level over 140 days, in the daily, 2-weekly, and 4-weekly groups, respectively. *Squares* and *triangles* represent D2 and D3 groups, respectively. *Circles* and *cross marks* represent combined D2/D3 and placebo groups, respectively. **d**, **e**, and **f**, data represent adjusted mean (SE) 25(OH)D AUC_140_ in the daily, 2-weekly, and 4-weekly groups, respectively

Figure 2a to c depicts mean 25(OH)D level from day 0 to day 140 in the eight groups. The concentration-time curve in the placebo group was rather flat with a maximum mean increase in 25(OH)D level of 6.2 compared to 27.5 to 37.3 nmol/L in the seven active-treatment groups. At day 140, the mean increase in in the active-treatment groups was 28.6 (16.3) compared to 3.3 (11.6) nmol/L in the placebo group. The curve flattened around day 70 in the daily groups and around day 112 in the 2-weekly groups. In the 4-weekly groups, it showed consistent fluctuation (mean differences around 6.6 and 5.2 nmol/L in D2 and D3 groups, respectively) with peaks and troughs, 2 weeks and 4 weeks after dosing, respectively. Interestingly, there was no further increase in peak 25(OH)D level after day 70 in the D2 4-weekly group, however, peak 25(OH)D level appeared to continue to increase over the duration of the study. The curves were not grossly separable in the three daily active treatment groups; however, they were clearly separable in the two 2-weekly groups and the two 4-weekly groups, with the groups assigned to D3 maintaining higher levels than the groups assigned to D2.

Analysis of 25(OH)D AUC_140_ showed significant effect of treatment group (*p* < 0.001) and baseline 25(OH)D level (*p* < 0.001) but not sex (*p* = 0.38) or BMI (*p* = 0.14). Figure 2d to f shows adjusted mean (SE) 25(OH)D AUC_140_ in the eight groups. All active treatment groups had significantly higher 25(OH)D AUC_140_ than the placebo group (40 to 55%) with mean difference ranging from 2530.4 nmol.d/L (CI, 1741.3 to 3319.6) in D3 daily group to 3503.3 nmol.d/L (CI, 2711.9 to 4294.6) in D3 2-weekly group. Adjusted mean 25(OH)D AUC_140_ was significantly lower in D3 daily group compared to D3 2-weekly group (mean difference −972.8 nmol.d/L (CI, −1751.1 to −194.6, *p* = 0.02) and D3 4-weekly group (mean difference −896.4 nmol.d/L (CI, −1710.2 to −82.6, *p* = 0.03). It was significantly higher in D3 2-weekly group compared to daily D2/D3 (mean difference 852.4 nmol.d/L (CI, 85.9 to 1618.9, *p* = 0.03) and D2 4-weekly group (mean difference 897.5 nmol.d/L (CI, 93.9 to 1701.1, *p* = 0.03). The increase in 25(OH)D level between days 0 and 140 correlated negatively with day zero 25(OH)D level (*r* = −0.21, *p* = 0.001). There was no significant difference in day 140 serum calcium level or urinary calcium, phosphate, or creatinine levels among the 8 groups (*p* = 0.58, to 0.98, adjusted for day zero value and sex).

**Fig. 3:**
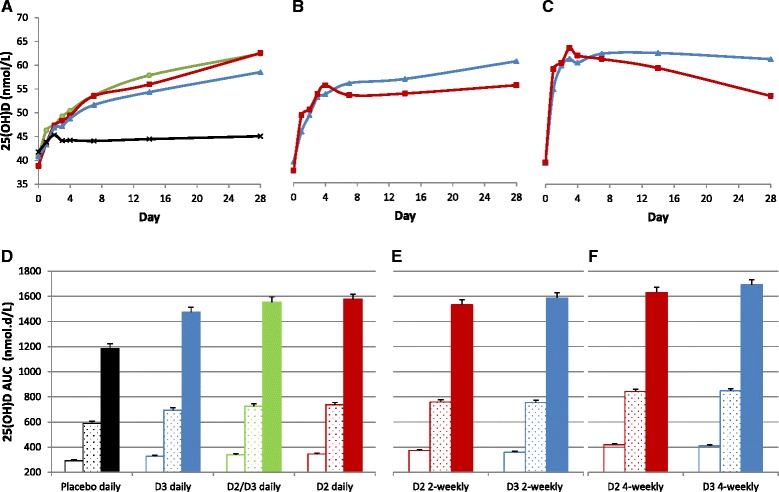
**a**, **b**, and **c**, data represent mean total 25-hydroxyvitamin D (25(OH)D) level over the first 28 days of the study, in the daily, 2-weekly, and 4-weekly groups, respectively. *Squares* and *triangles* represent D2 and D3 groups, respectively. *Circles* and *cross marks* represent combined D2/D3 and placebo groups, respectively. **d**, **e**, and **f**, data represent adjusted mean (SE) 25(OH)D AUC_7_ (*open bars*), AUC_14_ (*dotted bars*), and AUC_28_ (*closed bars*) in the daily, 2-weekly, and 4-weekly groups, respectively

Figure 3a to c depicts mean 25(OH)D level from day 0 to day 28 in the eight groups. Adjusted mean 25(OH)D level was not significantly different from the placebo group until day 2 in the D2 daily (*p* = 0.005) and until day 3 in the D3 daily (*p* = 0.02) and the D2/D3 daily (*p* = 0.001) groups. However, it was significantly different from day 1 in the D2 and D3 2-weekly and 4-weekly groups (*p* < 0.001 to 0.005). At day 4, adjusted mean 25(OH)D level was significantly lower in the D2 and D3 daily groups compared to the corresponding 2-weekly groups (*p* = 0.02 and 0.006, respectively) and in the D2 and D3 2-weekly groups compared to the corresponding 4-weekly groups (*p* = 0.03 and *p* = 0.004, respectively). At day 28, adjusted mean 25(OH)D level was higher in D3 4-weekly group compared to D2 4-weekly group (mean difference 6.4 nmol/L (CI, 1.5 to 11.3, *p* = 0.01)) and in D3 2-weekly group compared to D2 2-weekly group (mean difference 4.5 nmol/L (CI, −0.4 to 9.5, *p* = 0.07)). On the other hand, 25(OH)D level was significantly higher in D2 daily group compared to D3 daily group (mean difference 6.2 nmol/L (CI, 1.4 to 11.0, *p* = 0.01)).

Analysis of 25(OH)D AUC_7_, AUC_14_, and AUC_28_ showed significant effect of treatment group (*p* < 0.001), baseline 25(OH)D level (*p* < 0.001), and BMI (*p* = 0.002 to 0.004) but not sex (*p* = 0.55 to 0.85). Figure 3d to f depicts adjusted mean (SE) 25(OH)D AUC_7_, AUC_14_, and AUC_28_ in the eight groups. Adjusted mean 25(OH)D AUC_7_ was significantly (*p* < 0.001) higher in the active-treatment groups compared to the placebo group (mean difference ranging from 36.0 nmol.d/L (CI, 13.2 to 58.7) to 126.9 nmol.d/L (CI, 104.0 to 149.7). In the D3 treated groups, adjusted means of 25(OH)D AUC_7,_ AUC_14,_ and AU_28_ were significantly lower in the daily group compared to the 2-weekly (*p* = 0.005 to 0.04) and the 4-weekly (*p* < 0.001) groups. In contrast, in the D2 treated groups, although adjusted mean 25(OH)D AUC_7_ was significantly lower in the daily group compared to the 2-weekly and 4-weekly groups (*p* = 0.02 and *p* < 0.001, respectively), adjusted mean 25(OH)D AUC_14_ was significantly lower only in the daily group compared to the 4-weekly group, and adjusted mean 25(OH)D AUC_28_ was not significantly different in the daily group compared to the 2-weekly or 4-weekly groups (*p* = 0.42 and *p* = 0.36, respectively). There was significant negative correlation between 25(OH)D AUC_7_, AUC_14_, and AUC_28_ and BMI in the D2 treated groups (*r* = −0.25, *p* = 0.01 for all) but not in D3 treated groups (*r* = −0.10, *p* = 0.32 to 0.36). In summary, the data suggest that in the long term (20 weeks), D3 2-weekly followed by D3 4-weekly and D2 daily regimens may be superior in raising 25(OH)D level. However, in the first few weeks of treatment, the 4-weekly regimens followed by 2-weekly regimens appear to be superior to all daily regimens. Further, D3 2-weekly and 4-weekly regimens appear to be consistently superior to the corresponding D2 regimens, and D2 daily regimen appears to be consistently superior to D3 daily regimen. Finally, the increase in 25(OH)D level appears to be inversely related to BMI (mainly short term after D2 treatment) and to baseline 25(OH)D level.

### Differential effects of vitamin D regimens on 25(OH)D2 and 25(OH)D3 levels

**Fig. 4:**
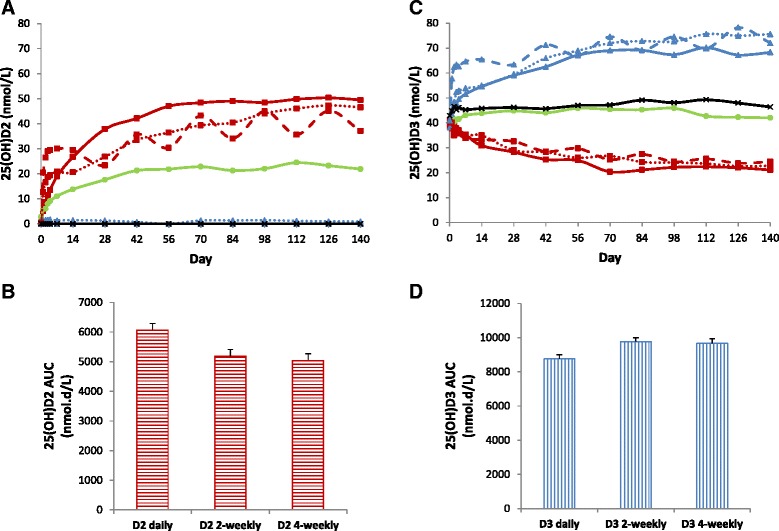
**a** and **c**, data represent mean 25-hydroxyvitamin D2 (25(OH)D2) and 25(OH)D3 levels, respectively, over 140 days. *Squares* and *triangles* represent D2 and D3 groups, respectively. *Circles* and *cross marks* represent combined D2/D3 and placebo groups, respectively. *Solid, doted*, and *interrupted lines* represent daily, 2-weekly, and 4-weekly groups, respectively. **b** and **d**, data represent adjusted mean (SE) 25(OH)D2 AUC_140_ in the D2 treated groups and 25(OH)D3 AUC_140_ in the D3 treated groups, respectively

Figure 4a shows mean 25(OH)D2 level from day 0 to day 140 in the eight groups. Analysis of 25(OH)D2 AUC_140_ showed significant effect of treatment group (*p* < 0.001) and baseline 25(OH)D2 level (*p* < 0.001) but not BMI (*p* = 0.18) or sex (*p* = 0.28). Figure 4b depicts adjusted mean (SE) 25(OH)D2 AUC_140_ in the three D2 treated groups. Adjusted mean 25(OH)D2 AUC_140_ was significantly higher (17 and 20%) in D2 daily group compared to D2 2-weekly group (mean difference 881.5 nmol.d/L (CI, 257.0 to 1506.1, *p* = 0.006)) and D2 4-weekly group (mean difference 1029.2 nmol.d/L (CI, 410.5 to 1648.0, *p* = 0.001)), with no significant difference between the 2-weekly and 4-weekly groups (*p* = 0.65). In contrast, adjusted mean 25(OH)D2 AUC_7_ and AUC_14_ were significantly lower in the D2 daily group compared to D2 2-weekly group (mean difference −42.0 nmol.d/L (CI, −56.5 to −27.6, *p* < 0.001) and −38.5 nmol.d/L (CI, −70.6 to −6.5, *p* = 0.02), respectively) and to D2 4-weekly group (mean difference −91.4 nmol.d/L (CI, −105.7 t0 -77.2, *p* < 0.001) and −136.9 nmol.d/L (CI, −168.0 to −105.8, *p* < 0.001), respectively). Further, 25(OH)D2 AUC_7_, AUC_14_, and AUC_28_ were significantly higher in D2 4-weekly group compared to 2-weekly group (*p* < 0.001, *p* < 0.001, and *p* = 0.01, respectively). Furthermore, 25(OH)D2 AUC_7_, AUC_14_, and AUC_28_ were significantly associated with BMI (*p* < 0.001, *p* < 0.001, *p* = 0.001, respectively) and sex (*p* = 0.003, *p* = 0.02, *p* = 0.046, respectively).

Figure 4c shows mean 25(OH)D3 level from day 0 to day 140 in the eight groups. Analysis of 25(OH)D3 AUC_140_ showed significant effect of treatment group (*p* < 0.001), baseline 25(OH)D3 level (*p* < 0.001), but not sex or BMI (*p* = 0.55). Figure 4d depicts adjusted mean (SE) 25(OH)D3 AUC_140_ in the three D3 treated groups. Adjusted mean 25(OH)D3 AUC_140_ was significantly lower in the D3 daily group compared to D3 2-weekly group (mean difference −1002.2 nmol.d/L (CI, −1641.4 to −363.0, *p* = 0.002)) and D3 4-weekly group (mean difference −910.5 nmol.d/L (CI, −1577.4 to −243.5, *p* = 0.008)), with no significant difference between the 2-weekly and 4-weekly groups (*p* = 0.79). These differences started early on; adjusted mean 25(OH)D3 AUC_7_, AUC_14_, and AUC_28_ were significantly lower in D3 daily group compared to D3 2-weekly group (*p* < 0.001, *p* = 0.003, and *p* = 0.01, respectively) and D3 4-weekly group (*p* < 0.001, *p* < 0.001, and *p* < 0.001, respectively), and adjusted mean 25(OH)D3 AUC_7_, AUC_14_, and AUC_28_ were significantly higher in the D3 4-weekly group compared to 2-weekly group (*p* < 0.001, *p* = 0.001, and *p* = 0.046, respectively). Further, 25(OH)D3 AUC_7_, AUC_14_, and AUC_28_ were not associated with BMI (*p* = 0.32 to 0.61) or sex (*p* = 0.18 to 0.34).

**Fig. 5:**
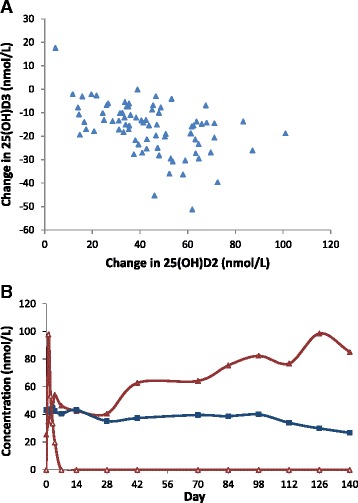
**a**, Data show correlation between the changes (day 140 - day 0) in 25(OH)D3 levels and 25(OH)D2 levels in the three D2 treated groups (*r* = −0.48, *p* < 0.001). **b**, levels of D3 (*open triangles*), 25(OH)D2 (*closed squares*), and 25(OH)D3 (*closed triangles*) are depicted over 140 days in one participant from the D3 2-weekly group who happened to have measurable 25(OH)D2 at day zero

Interestingly, in the D2/D3 group, 25(OH)D2 level increased to about one third to one half the levels in the three D2 treated groups (Fig. 4a, however, 25(OH)D3 did not increase (Fig. 4c. Further, in the three D2 treated groups, 25(OH)D3 level progressively and consistently decreased over the course of the study (Fig. 4c. It declined from a mean of 39.2 nmol/L at day 0 to 33.0, 31.6, 28.6, and 22.9 at days 7, 14, 28, and 140, respectively. To further explore the effect of D2 treatment on 25(OH)D3 level, we examined the correlation between the changes (day 140 minus day 0) in 25(OH)D3 level and 25(OH)D2 level in the three D2 treated groups. As shown in Fig. 5a, there was significant negative correlation (*r* = −0.48, *p* < 0.001). However, the change in 25(OH)D3 level also correlated negatively with day zero 25(OH)D3 level (*r* = −0.58, *p* < 0.001) and with day 140 25(OH)D levels (*r* = −0.32, *p* = 0.003). Of note, in one participant who started with measurable 25(OH)D2 level and received D3 2-weekly, 25(OH)D2 level decreased by 16.4 nmol/L between days 0 and 140 (Fig. 5b). In comparison, the mean decrease in 25(OH)D3 level in the three D2 treated groups was 16.4 nmol/L (CI, 14.0 to 18.7, *p* < 0.001), suggesting that D3 treatment may also induce a decline in 25(OH)D2 level of the same magnitude.

### Differential effects of vitamin D regimens on D2 and D3 levels

**Fig. 6:**
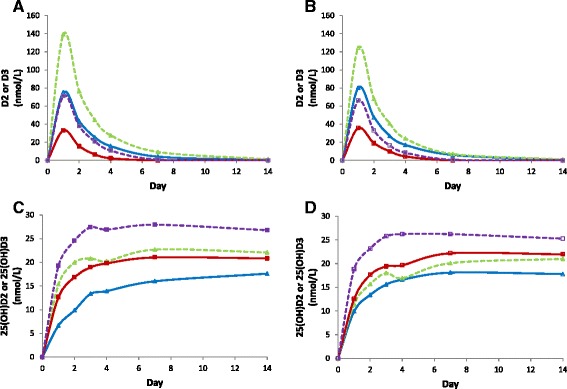
**a**, data represent mean D3 (*closed triangles*) and D2 (*closed squares*) levels in the 4-weekly (interrupted lines) and 2-weekly (*solid lines*) groups. **b**, data represent mean D3 (*open triangles*) and D2 (*open squares*) levels in females (*interrupted line*) and males (*solid lines*) in the 2-weekly and 4-weekly groups. C, data represent mean 25(OH)D3 (*closed triangles*) and 25(OH)D2 (*closed squares*) levels in the 4-weekly (*interrupted lines*) and 2-weekly (*solid lines*) groups after subtracting day zero levels. D, data represent mean 25(OH)D3 (*open triangles*) and 25(OH)D2 (*open squares*) levels in females (*interrupted lines*) and males (*solid lines*) in the 2-weekly and 4-weekly groups after subtracting day zero levels

At day zero, D2 and D3 levels were undetectable or only traceable in all the 8 groups. D2 and D3 levels were quantifiable only during the first week of treatment in the 2-weekly and 4-weekly groups. The following analysis is therefore restricted to the 2-weekly and 4-weekly groups. D2 and D3 concentration-time curves in the 2-weekly and 4-weekly groups are shown in Fig. 6a and b. As expected mean D2 and D3 levels were twice as high in the 4-weekly groups compared to the 2-weekly groups. Interestingly, mean D3 levels in the D3 treated groups were higher than mean D2 levels in the corresponding D2 treated groups (Fig. 6a). In the D2 treated groups, analysis of D2 AUC_7_ showed significant effect of treatment group (*p* < 0.001), sex (*p* = 0.01), and BMI (*p* = 0.001). In the D3 treated groups, analysis of D3 AUC_7_ showed significant effect of treatment group (*p* < 0.001), sex (*p* < 0.001), but not BMI (*p* = 0.06). Adjusted mean D2 AUC_7_ was 200% higher in the 4-weekly group compared to 2-weekly group with a mean difference of 100.2 nmol.d/L (CI, 62.3 to 138.0, *p* < 0.001), and adjusted mean D3 AUC_7_ was 90% higher in the 4-weekly group compared to 2-weekly group with a mean difference of 155.7 nmol.d/L (CI, 109.8 to 201.6, *p* < 0.001). Adjusted mean D3 AUC_7_ was 243% higher than D2 AUC_7_ in the 2-weekly groups (mean (SE) 172.2 (16.1) vs 50.2 (13.5) nmol.d/L) with a mean difference of 122.0 nmol.d/L (CI, 79.8 to 164.1, *p* < 0.001) and 118% higher in the 4-weekly groups (mean (SE) 327.9 (16.4) vs 150.4 (13.4) nmol.d/L) with a mean difference of 177.5 nmol.d/L (CI, 135.3 to 219.8, *p* < 0.001). Figure 6b shows that mean D2 levels in D2 treated groups and mean D3 levels in D3 treated groups are (64 and 55%, respectiv1ely) higher in females than males. Adjusted mean difference in AUC_7_ was 48.5 nmol.d/L (CI, 10.3 to 86.6, *p* = 0.01) for D2 and 107.7 nmol.d/L (CI, 61.5 to 154.0, *p* < 0.001) for D3. Interestingly, BMI correlated significantly with D2 AUC_7_ (*r* = −0.27, *p* = 0.03) but not D3 AUC_7_ (*r* = −0.05, *p* = 0.67).

To explore the reasons for the differential effects of sex and D-type on D2 and D3 levels, we examined 25(OH)D2 levels in the D2 treated groups and 25(OH)D3 levels in the D3 treated groups after subtracting the corresponding baseline levels. As shown in Fig. 6c, mean 25(OH)D2 levels in the D2 treated groups were higher than mean 25(OH)D3 levels in the corresponding D3 treated groups. Adjusted mean (SE) 25(OH)D2 AUC_7_ was 39% higher than adjusted mean 25(OH)D3 AUC_7_ in the 2-weekly groups (mean (SE) 107.9 (8.9) vs 77.4 (7.7) nmol.d/L) with a mean difference of 30.5 nmol.d/L (CI, 7.2 to 53.9, *p* = 0.01) and 31% higher in the 4-weekly groups (mean (SE) 163.2 (8.9) vs 124.5 (8.0) nmol.d/L) with a mean difference of 38.7 nmol.d/L (CI, 14.8 to 62.5, *p* < 0.002). This together with higher D3 levels in the D3 treated groups compared to D2 levels in the D2 treated groups as shown above, suggest faster 25-hydroxylation of D2 compared to D3. However, faster 25-hydroxylation could not account for all the observed difference between D2 and D3 levels.

As shown in Fig. 6d, mean 25(OH)D2 levels in the D2 treated groups and mean 25(OH)D3 levels in the D3 treated groups were higher in females compared to males. Adjusted mean (SE) 25(OH)D2 AUC_7_ was 28% higher in females compared to males (152.2 (8.1) vs 118.9 (9.6) nmol.d/L) with a mean difference of 33.4 nmol.d/L (CI, 8.2 to 58.5, *p* = 0.01). Adjusted mean (SE) 25(OH)D3 AUC_7_ was 13% higher in females compared to males (106.9 (7.2) vs 95.0 (8.6) nmol.d/L) with a mean difference of 11.9 nmol.d/L (CI, −10.8 to 34.6, *p* = 0.3). In retrospect, the significant difference between females and males in 25(OH)D2 levels in the 2-weekly and 4-weekly groups was found to be present at day 140; mean (SE) 25(OH)D2 AUC_140_ was 5509.0 (274.2) vs 4526.5 (313.5) nmol.d/L with a mean difference of 982.5 nmol.d/L (CI, 137.6 to 1827.3, *p* = 0.02). This together with higher D2 levels in D2 treated groups and D3 levels in the D3 treated groups in females compared to males as shown above, suggest higher D2 and D3 circulation availability in females compared to males. Finally, BMI correlated significantly with 25(OH)D2 AUC _7_ (*r* = −0.34, *p* = 0.006) but not 25(OH)D3 AUC_7_ (*r* = −0.11, *p* = 0.38).

## Discussion

The primary aim of this randomized placebo-controlled, partially-blinded study on 269 healthy adults with mean 25(OH)D of 39.8 (11.9) nmol/L was to evaluate the relative efficacy of equi-unit D2 and D3 oral supplements given daily, 2-weekly, or 4-weekly in raising 25(OH)D level over 20 weeks. Predetermined secondary aims included comparing D2, D3, 25(OH)D2, and 25(OH)D3 levels. The primary outcome measure was adjusted area-under-the-curve between days 0 and 140 (AUC_140_)_._ The main results were: 1) in the long term (20 weeks), the D3 2-weekly followed by D3 4-weekly and D2 daily regimens were superior in raising 25(OH)D levels. In the first few weeks of treatment, however, the 4-weekly followed by 2-weekly regimens were superior to all daily regimens. 2) D3 2-weekly and 4-weekly regimens were consistently superior to the corresponding D2 regimens; however, D2 daily regimen was consistently superior to D3 daily regimen. 3) 25(OH)D2 level was significantly higher in the daily compared to the 2-weekly and 4-weekly D2-treated groups, whereas, 25(OH)D3 level was lower in the daily compared to the 2-weekly and 4-weekly D3-treated groups 4) The increase in 25(OH)D level was inversely related to baseline level, however, its inverse relation to BMI appeared to be D-type and time dependent (mainly short time after D2 treatment). 5) Daily, 2-weekly, and 4-weekly D2 regimens were associated with a similar and significant decrease in 25(OH)D3 level that correlated with the increase in 25(OH)D2 level and baseline 25(OH)D level, in one participant with measurable baseline 25(OH)D2 level, D3 caused a similar decrease in 25(OH)D2 level, while in the D2/D3-treated group, 25(OH)D3 level didn’t increase. 6) The increases in D3 level in the 2-weekly and 4-weekly D3 treated groups were higher than the increases in D2 level in the corresponding D2 treated groups, the opposite was true for 25(OH)D2 and 25(OH)D3 levels. 7) Females had higher increases in D3, D2, 25(OH)D2, and 25(OH)D3 levels than males. 8) D treatment was associated with significant increases in urinary calcium and creatinine levels but not calcium/creatinine ratio.

All of the seven D supplement regimens in our study significantly increased 25(OH)D. At day 140, mean increase was 3.3 nmol/L in the placebo group and 28.6 nmol/L in the active-treatment groups. Although comparison is difficult because the dose–response curve is curvilinear, with an average of 1786 IU/day, this translates into an increase of about 16 nmol/L per 1000 IU, which is consistent with previous observations [8, 21, 26, 27, 36, 40]. In men with baseline 25(OH)D of 70 nmol/L, it was estimated that the increase in 25(OH)D level is about 17.5 nmol/L per 1000 IU daily D3 dose [40]. A review of recordings of 17,614 healthy adults participating in a preventive health program found an average increase of 12 nmol/L per 1000 IU for daily dosing interval of 0 to 1000 IU [21]. In a multicenter, retrospective data extraction study, an average daily dose of 2700 IU D3 increased 25(OH)D by 11.8 nmol/L per 1000 IU [27]. It is to be noted that the recovered content of the capsules in our study was about 90% of the label claim and that compliance with study medication was 98.4 to 100%. In our study, the increase in 25(OH)D level plateaued around days 70 and 112 in the daily and 2-weekly groups, respectively. Time to plateau ranged from 5 weeks to five months in previous studies [40, 41]. Due to our relatively frequent sampling, we were able to observe clear fluctuations in 25(OH)D levels when measured 2 weeks and 4 weeks after dosing, which may have clinical implication in term of monitoring response to therapy. Interestingly, the fluctuations were more pronounced with D2 dosing, consistent with shorter half life of 25(OH)D2 [4, 7, 42].

Our finding that D3 is superior to D2 in raising 25(OH)D level when given 2-weekly or 4-weekly is consistent with the published literature. The superiority of D3 was seen in studies that used 50,000 IU daily [5] or weekly, [4] a bolus of 300,000 [42] or 50,000 IU, [7] and a bolus of 10,000,000 IU in cows, [43] but not in studies that used 400 IU daily, [8, 9] 1000 IU daily, [29, 36] or 2000 IU daily [10]. Nevertheless, it was also reported with daily doses of 4000 IU for 14 days [11]. A 2012 meta-analysis found that D3 is more potent than D2, interestingly the difference was significant in the 4 RCTs (48 patients) that used bolus oral or intramuscular doses but not in the 6 RCTs (146 patients) that used daily supplements [24]. The interaction between D-type and dosing schedule was clearly shown in this study; while daily D3 was less efficient than 2-weekly and 4-weekly D3 in raising 25(OHD3, daily D2 was superior to 2-weekly and 4-weekly D2 in raising 25(OH)D2 levels. It is to be noted that the formulation of the capsules in our study was based on the common unitage that 1 IU equals 25 ng crystalline D2 or D3. Since the molecular weights of D2 and D3 are 384 and 396, respectively, 25 ng D3 would be equivalent to 25.78 ng, [28] thus the potency of D2 may have been underestimated by about 3% if one considers molar equivalence rather than weight equivalence in determining potency in IUs. Our results suggest that for long term results, D2 is best given daily while D3 is best given 2-weekly. However, the 4-weekly followed by the 2-weekly (D2 or D3) regimens are clearly superior in in rapidly raising 25(OH)D levels.

We observed consistent decrease in 25(OH)D3 levels in D2 treated groups. This was observed in most [8, 33, 44] but not all [29] previous studies that fractionated 25(OH)D levels. In a meta-analysis of RCTs on the effect of UV-exposed mushrooms consumption, the increase in 25(OH)D2 level was associate with a decrease in 25(OH)D3 level [35]. Further, 1000 IU D3 daily for 11 week did not change 1,25(OH)_2_D3 level, while 1000 IU D2 daily increased 1,25(OH)_2_D2 level by 7.4 pg/ml and decreased 1,25(OH)_2_D3 level by 9.9 pg/ml [36]. A similar decrease in 1,25(OH)_2_D3 was seen in response to 4000 IU D2 daily for 8 weeks [44]. Several observations from our study may shed light on the mechanism(s) underlying these observations. We found that the D2-induced decrease in 25(OH)D3 level was similar in the daily, 2-weekly, and 4-weekly groups, that it was correlated with the increase in 25(OH)D2 level, baseline 25(OH)D level, and day 140 25(OH)D level, that there was no change in 25(OH)D3 level in the group treated with a combination of D2 and D3, and that when 25(OH)D2 level is measurable (one case), D3 treatment resulted in a similar decrease in 25(OH)D2 level. These observations suggest that the D2-induced decrease in 25(OH)D3 level may more related to the resulting 25(OH)D level rather than being specific to D2 treatment. In fact, in one study, 400 and 1000 IU D3 daily for one year resulted in an increase in 25(OH)D3 level with a concomitant decrease in 25(OH)D2 level [34]. Interestingly, in a crossover study on high-yielding dairy cows, pre-administration of 10,000,000 IU of D3 significantly reduced 25(OH)D2 response to 10,000,000 IU of D2 [43]. It may be that there is a regulatory mechanism that increases the disposal of 25(OH)D in response to increases in its level [20] and that it has been observed with D2 treatment mainly because study participants commonly don’t have measurable 25(OH)D2 levels. Since there was essentially no change in 25(OH)D3 level in the group that received combination of 1000 IU D2 and 1000 IU D3, it appears that, in a setting similar to our study (baseline 25(OH)D level around 40 nmol/L and average dose of 1800 IU daily), an amount of 25(OH)D that can be produced by 1000 IU intake is disposed daily. If such a mechanism really exists it can be exploited in defining normal 25(OH)D levels.

Consistent with the above interpretation and with previous studies, [9, 11–16, 26, 35] we found significant negative correlation between baseline 25(OH)D level and response to treatment. A recent review found that 17 out of 20 studies documented such correlation (3 studies had inadequate sample size and variation in baseline level), which may explain up to 20% of response variation [13]. A recent systematic review of studies that used modest daily doses of D3 (200 to 800 IU), also found negative correlation, albeit not significant [26]. The negative correlation together with the non-linear response in 25(OH)D level to increasing doses of D [6, 16, 20, 21] again suggest a regulatory step mechanism [20]. In our study, the significant negative correlation between baseline and increment 25(OH)D level was first seen at day 28 (when 25(OH)D level was 59.1 (14.7) nmol/L), suggesting a threshold effect. Interestingly, pooling data of 3 RCTs, subjects with single nucleotide polymorphisms (linked to D binding proteins and 25-hydroxylase) that are associated with the lowest baseline 25(OH)D level had the smallest increase in 25(OH)D level (32). Thus in some subjects, low baseline 25(OH)D level may reflect a genetic potential rather than lifestyle influence and may be associated with lower rather than higher increment in 25(OH)D level.

Higher BMI/body fat percentage was associated with smaller response to D supplement in several studies [12–14, 18–21, 45]. BMI may be a better predictor than absolute weight [21] and was suggested as the most powerful response predictor to D supplement [19]. Up to 34.5% of response variation may be related to BMI, more apparently with higher D doses [13]. In a large retrospective study, mean increases in 25(OH)D level were 28.7, 23.6, and 20.1 nmol/L with BMI <25, 25–29, or ≥ 30 kg/m2, respectively [18]. Nevertheless, not all studies showed such negative association [13]. This may be due to the fact that higher BMI is also associated with baseline lower 25(OH)D levels, [17, 45] which is itself positively associated with response to D supplement. In our study, BMI was a significant response predictor to D2 but not D3 and only during the first 4 weeks of treatment, suggesting two additional potential modifiers of the relationship between BMI and 25(OH)D response to D supplement, D-type and time of assessment. The mechanisms underlying the association between BMI and response to D supplement are not clear. It was suggested that D and 25(OH)D may be trapped in access adipose tissue, as 25(OH)D is released with weight loss 1–6 months following bariatric surgery [46, 47]. However, total body fat storage may account for only 17% of the administered dose (extrapolated from subcutaneous fat), [4] and D and 25(OH)D may be also deposited in liver, muscle, and skin as shown in animal studies, suggesting that volume dilution may play a role [20]. The relatively lower affinity of D binding protein to D2 and 25(OH)D2 [24, 28] makes them more accessible to extra-vascular tissues, which may explain our finding.

We found that the increase in D3 level was 2–3 fold higher than the increase in D2 level (in the 2-weekly and 4-weekly treated groups). Few studies examined D2 and D3 after D2 and D3 supplementation [4, 43]. After similar doses of D2 and D3 (50,000 IU weekly for 12 weeks), subcutaneous fat D3 storage was 2 times higher than D2 storage [4]. Equivalent pharmaceutical doses of D3 and D2 in cows increased D3 level more than D2 level, respectively [43]. The difference may be related to the different structure of D2 and D3 side chains, theoretically causing differential absorption, binding to D binding proteins, inactivation by 24-hydroxylation, or activation by 25-hydroxylation. Absorption is not likely to be involved as studies of tritium-labeled D2 and D3 in healthy subjects found similar recoveries after oral dosing, however, D binding protein has double association constant to D3 compared to D2, and in vitro, mitochondrial 25-hydroxylase is 5 times faster for D3 compared to D2 [11, 24, 28]. It is to be noted that most of the ingested D is not converted to 25(OH)D; an RCT found that oral 25(OH)D3 is 4–5 more potent than D3 in raising 25(OH)D3 levels, [41] Interestingly, in our study, 25(OH)D2 levels were higher than 25(OH)D3 levels, suggesting that the lower D2 levels were due, at least in part, to higher D2 accessibility to the 25-hydroxylase enzyme because of lower affinity to D binding proteins. It is of note that the combination of D3 AUC_7_ and 25(OH)D3 AUC_7_ was about 50% higher than the combination of D2 AUC_7_ and 25(OH)D2 AUC_7_; indicating that a mechanism other than faster 25-hydroxylation (such as higher accessibility of D2 and 25(OH)D2 to extra-vascular tissues and faster degradation) is also involved. These mechanisms may apply only for schedules using high doses as 25(OH)D AUC_140_ was higher in D2 daily than D3 daily treatment.

The higher levels of D3 compared to D2 may have important implication regardless of their impact on serum 25(OH)D level. Because of lower affinity to D binding protein, D2 and D3 have more cellular accessibility than 25(OH)D2 and 25(OH)D3 (except for the kidney, parathyroid gland, and placenta, where the megalin-cubulin system is expressed) and may have important physiological roles in breast milk and as substrates for many tissues [2].

We found that females had significantly larger (60, 55, 28%) adjusted AUC_7_ than males for D2, D3, and 25(OH)D2 levels (and larger 13%,but not significant increase in 25(OH)D3 level). Females also had significantly 22% larger adjusted 25(OH)D2 AUC_140._

The results suggest about 49% better bioavailability of both D2 and D3 in females. Sex effect on response to D supplement has not been directly studied before. However, it is of note that D binding proteins are higher in females than males, in premenopausal women compared to postmenopausal women, in pregnant women, in women on oral contraceptives, [22] and in postmenopausal women hormone replacement therapy [23]. Further, estrogens increase hepatic 25-hydroxylation of D and the impact of D binding protein on response to D treatment may be partly D-type dependent [13]. The observed sex differences may be related to higher D binding protein and faster 25-hydroxyaltion in females; although a sex difference in D absorption rate cannot be excluded. It is also possible that higher body fat and lower baseline 25(OH)D levels in females may play a role.

In agreement with previous studies using even higher doses of D, [6, 48, 49] none of our participants developed hypercalcemia or hypercalciuria. In fact, the increase in urinary calcium/creatinine ratio was not significant. Nevertheless, the calcium/creatinine ratio may be misleading as there was significant increase in both calcium and creatinine urinary excretion. An increase in creatinine generation and urinary excretion has been described in patients with chronic kidney disease treated with vitamin D receptor activator, paricacitol [50]. The increase in creatinine excretion associated with D treatment casts doubt on the usefulness of ratios that include urinary creatinine (such as albumin/creatinine and calcium/creatinine) in evaluating the effect of D treatment on kidney function [51, 52] or D intoxication [53].

The strengths of this study include using repeated measurements, having a placebo arm, and ability to study several active-treatment regimens simultaneously, which enabled observing small changes and uncovering mechanistic insights. They also include effective randomization and concealment, partial blinding, frequent follow up to strengthen and verify compliance and verification of D capsule content across the study period.

### Limitations

The interpretation of the results of this study may be limited by its sample size, 15% follow up loss, lower compliance with daily compared to 2-weekly and 4-weekly regimens, capsule content that is lower than label claim, and capsule formulation based on weight equipotency of D2 and D3. The rate of follow up loss was similar across the groups, the characteristics of participants who completed the study were similar to those of the entire cohort, incompliance rate was 1 to 1.6% in the daily groups, and the discrepancy between capsule content and label claim was similar across the capsules; thus these factors would not be expected to affect the main findings of the study. Although the incompliance rate was low, it was measured by capsule count, which may not be reliable. Thus the lower dose–response observed with daily D3 treatment compared to 2-weekly and 4-weekly D3 treatments could be explained at least in part by incompliance. However, such explanation is not likely given our observation that the dose–response was higher with daily D2 treatment compared to 2-weekly and 4-weekly D2 treatments and the fact that assignment to daily D2 or D3 treatment was random and blinded. The lower capsule content implies that the observed increments in D and 25(OH)D levels may have been up to 10% higher. Further, the fact that the molecular weight of D2 is about 3% higher compared to D3 indicates that our study may have underestimated response to D2 treatment. Nevertheless, such difference would not be expected to change the conclusions of the study. Further, the strength (in terms of IU) of most currently available D supplements is based on the assumption of weight rather than molar equipotency of D2 and D3. Another limitation of the study is that our findings may not be generalizable to lower or higher doses of vitamin D, or to subjects with different baseline 25 (OH)D levels, with different demographics, or with co-morbidities. Also, since the study was exploratory in nature, we have conducted multiple comparisons (including ad hoc comparisons), which would increase the rate of type 1 error. Further, the study examined surrogate endpoints (vitamin D and hydroxyvitamin D levels) rather than clinical endpoints. Finally, due to our assay sensitivity, we were not able to measure D2 and D3 levels in the daily treated groups.

## Conclusions

We conclude that: 1) the effects of D2 and D3 supplements on 25(OH)D level may be dosing-schedule, time and sex dependent. In the long term, D2 appears to be most effective when given daily and D3 appears to be most effective when given 2-weekly. Further, females appear to mount larger 25(OH)D response to D2 than males. 2) The D2-associated reduction in 25(OH)D3 level appears to be related to the increase in 25(OH)D level rather than being D2-specific, as it is related to baseline 25(OH)D level and D3 treatment may be also associated with a reduction in 25(OH)D2 level. This together with the well known association between baseline 25(OH)D level and response to D treatment suggest a regulatory mechanism that may be exploited in fine tuning determination of normal 25(OH)D level. 3) D2 appears to be 25-hydroxylated faster than D3. 4) The association between BMI and response to D supplement may be more pronounced with D2 and during the first few weeks of treatment. 5) D2 and D3 level in response to treatment are higher in females compared to males. 6) D treatment is associated with an increase in urinary creatinine level, which makes assessment of D effect using ratios that include creatinine misleading.

## Abbreviations

1,25(OH)_2_D2: 1,25 diydroxyvitamin D2
1,25(OH)_2_D3: 1,25 diydroxyvitamin D3
25(OH)D: Total 25-hydroxyvitamin D (25(OH)D2 + 25(OH)D3)
25(OH)D2: 25-hydroxyvitamin D2
25(OH)D3: 25-hydroxyvitamin D3
ANCOVA: Analysis of co-variance
AUC_14_: Area-under-the-curve from day 0 to day 14
AUC_140_: Area-under-the-curve from day 0 to day 140
AUC_28_: Area-under-the-curve from day 0 to day 28
AUC_7_: Area-under-the-curve from day 0 to day 7
BMI: Body mass index
CI: Confidence interval
CV: Coefficient of variation (standard deviation/mean)
D: Vitamin D
D2: Vitamin D2 or ergocalcifrol
D3: Vitamin D3 or cholecaciferol
HPLC: High performance liquid chromatography
KFSH&RC: King Faisal Specialist Hospital and Research Center
